# Our Contemporaries

**Published:** 1913-03

**Authors:** 


### OUR CONTEMPORARIES.

---

Dr. Paul M. Pilcher, who has edited the *Long Island Medical Journal* during the first six years of its existence, retires to the associate staff. The February issue appears under the editorial control of Dr. Henry Goodwin Webster, to whom we wish the same degree of success that has attended Dr. Pilcher's administration.

Dr. R. B. H. Gradwohl, Editor of the *General Practitioner,* published in the February issue an interesting article on Laboratory Equipment, and completes the publication of a translation of Rudolph Allers' article on the Value and Significance of Recent Advances in Immunity, in the Practice of Psychiatry. The original appeared in the Zeitschrift für Chemotherapie, No. 10 and 11, 1912. The bibliography alone occupies eleven pages.

